# GMRS Oncological Prosthesis with a Porous Coating Collar: A Good Option for Revision of Aseptic Loosening in the Lower Extremity

**DOI:** 10.3390/jcm12030892

**Published:** 2023-01-23

**Authors:** Hairong Xu, Yuan Li, Feng Yu, Weifeng Liu, Lin Hao, Qing Zhang, Xiaohui Niu

**Affiliations:** Department of Orthopedic Oncology Surgery, Beijing Jishuitan Hospital, Peking University, Beijing 100871, China

**Keywords:** aseptic loosening, lower extremity, revision, function

## Abstract

Background: Revisions for oncological prosthesis are especially challenging due to the limited bone quantity and poor quality that the patients still possess. The aims of this study were to ask (1) what is the cumulative survival of the Global Modular Replacement System (GMRS) prosthesis after revision? and (2) what are the long-term functional outcomes of these patients? Methods: We retrospectively reviewed 16 patients who developed aseptic loosening of a lower extremity prosthesis. There were nine males and seven females with a mean age of 28 years (range, 14–55 years). The 5-year and 8-year survivorship of the prosthesis were calculated. Function outcome was evaluated according to the score of the Musculoskeletal Tumor Society (MSTS). Results: At a mean of 90 months follow-up (range, 52–118 months), the cumulative survival of all revision prosthesis was 94% at both 5 and 8 years. There were two prosthesis failures including one infection and one repeated aseptic loosening. At the last follow-up, except for the infection case, 93.3% (14/15) of the patients did not develop repeated aseptic loosening. The mean MSTS score was 27.7 (range, 24–30). Conclusions: GMRS prosthesis demonstrated significant satisfactory long-term outcomes for revisions of lower extremity oncological prosthesis.

## 1. Introduction

Amputation used to be the standard treatment for malignant bone tumors for quite a long time in history [1]. Advances in medical oncology, surgical technique, and reconstruction options have enabled limb-salvage surgery to be the most preferred method of treatment against amputation [2,3]. Although there are many reconstruction methods for the defects in the lower extremities after tumor resection, mega-prosthesis replacement has become the most widely used one currently [4,5]. However, with the increasing population and longer survival of the prosthetic patients, long-term reconstruction failures await most of them [6,7].

Aseptic loosening accounts for 19.1% of all failures and ranks as the second most common failure type [7]. Potential risk factors for aseptic loosening include remaining bone length, stem length, stem diameter, extracortical bone ingrowth, limb alignment, etc. [8,9,10]. Failures of the aseptic loosening mega-prosthesis leave surgeons with very few reconstruction options; moreover, sometimes, the treatment results in amputation [11]. The residual bone segment is usually short with poor quality [12]. As a result, the remaining bone segment may be sacrificed for a larger prosthesis reconstruction, such as total femur replacement for the original distal femur replacement [13]. This is the reason that revisions for aseptic mega-prosthetic loosening could be very challenging.

We need to change the prosthesis replacement strategy to maintain the remaining bone stock while creating a more durable revision outcome. Research by Bergin et al. suggested that the stem diameter independently predicts the aseptic loosening [9]. Patients with stable prosthesis had larger stem sizes and lower bone:stem ratios than those with loose implants. In this case, prosthesis with a short but large diameter stem is warranted. The Global Modular Replacement System (GMRSTM, Stryker, Kalamazoo, MI, USA) provides cemented stems in six styles including straight, curved, and long curved, each style with or without extra-cortical, porous-coated body sections. The extra-cortical porous-coated body section has a 4-cm replacement length. Although this prosthesis system is designed for primary reconstruction after tumor resection, we believe that the large-diameter (φ 13.15 mm) short stem (12.7 cm) with extra-cortical, porous-coated body sections are especially suitable for the revisions. To the best of our knowledge, this prosthesis, as indication for mega-prosthesis revision, has not been evaluated and reported until now.

Therefore, in this study, we asked (1) what is the cumulative survival of the prosthesis after revision? and (2) what are the long-term functional outcomes for patients with prosthesis?

## 2. Patients and Methods

Patients were retrieved from a prospectively collected database of JST Sarcoma & Bone Tumor Center [14,15]. Between 2009 and 2012, a total of 25 patients underwent revision surgery for mega-prosthesis aseptic loosening in the lower extremities in our hospital. Indications for revision to a GMRS mega-prosthesis included aseptic loosening, a minimum 17 cm length and 1 mm cortical thickness of the remaining bone segment evaluated using preoperative radiographic imaging including an X-ray of the whole lower extremities and a computerized tomography (CT) scan. Additional indications included no infection of the affected leg, no history of radiation in the lower extremities, a life expectancy greater than 10 years, no localized tumors, and no distant metastasis. Nine patients did not meet these indications or refused this prosthesis because of financial problems. Ethical approval for this study was obtained from our institution.

The remaining 16 patients received GMRS prosthesis revision exclusively for aseptic loosening. There were 9 males and 7 females with a mean age of 28 years (range, 14–55 years) at the time of revision. The most common primary diagnosis for tumor resection was conventional osteosarcoma (Table 1). All patients undergoing revision had finished the oncologic treatment at least 2 years ago. Eleven patients had their primary tumor resection and prosthesis replacement at our hospital. Five patients had their tumor resection and primary prosthesis replacement outside of our hospital and were referred to us for revision. Except for the 2 cases of proximal femur replacement, there were 8 rotating hinge knees and 6 constrained condylar knees for tumors around the knee including the distal femur and the proximal tibia.

**Figure 1:**
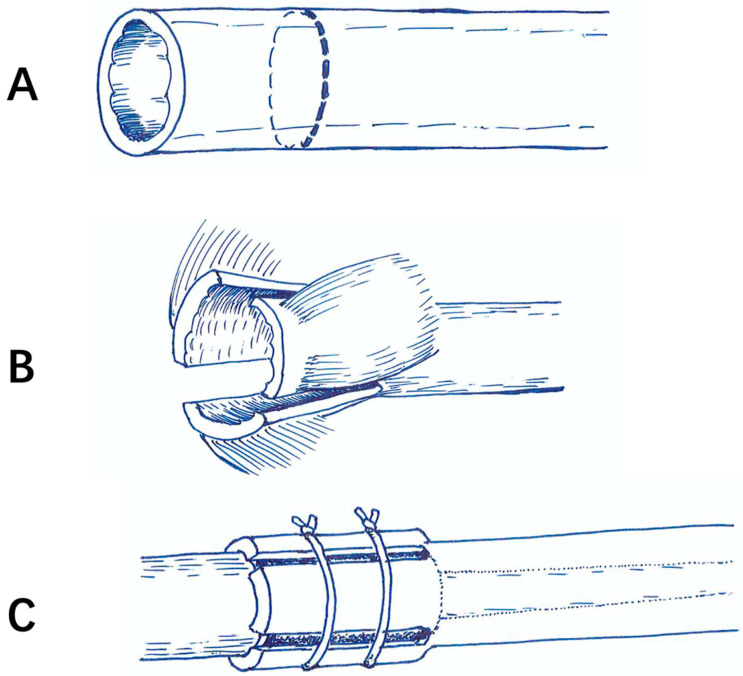
(**A**) After the cement was removed, the cortical pedicle was marked 4–5 cm away from the bone end. (**B**) The cortical pedicle was divided into three equal parts with a pendulum jigsaw. The procedure was carefully performed to keep the muscle attachments and periosteum. (**C**) The cortical pedicle autograft was affixed to this porous coating section with one or two wires to make a bone-bridging.

**Figure 2:**
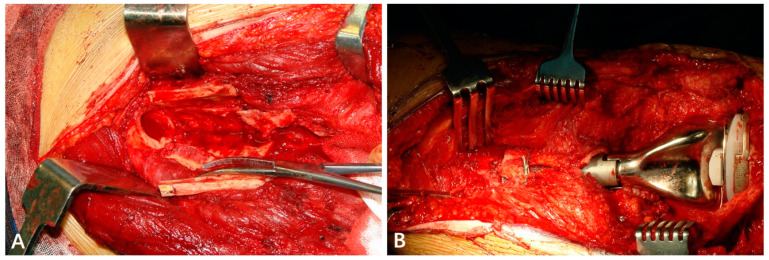
(**A**) In this case, the cortical bone was divided into five parts to facilitate the bone-bridging. The continuity of the cortical bone with the remaining bone was maintained in 4 of them. (**B**) The cortical bone-bridging was fixed to the porous coating section with one wire.

All patients were revised with a cemented GMRS stem, which was forged and 12.7 cm in length. The revision procedure included two steps in one stage. The first step was the removal of the old prosthesis and the second is the placement of the new prosthesis. All parts of the primary loosening prosthesis were removed; even the other part was not loosened. The cement from the primary prosthesis replacement was removed with the help of cement removal instruments as much as possible. The removal instruments included various sizes of reamer and osteotome, high-speed burr, flushing gun, etc. After the cement was removed, the medullary cavity was reamed until the surgeons encountered cortical “chatter”. The thickness of the remaining cortical bone should be at least 1 mm to hold the stem. The narrowest part of the medullary cavity should be reamed 1 mm larger than the stem diameter. For the selection of GMRS prosthesis stems of various diameter size, surgeons tried to choose one as large as possible. GMRS prosthesis is provided with a porous coating section over the shoulder region of the implant, which requires an autograft cortical bone to facilitate bone-bridging around this section (Figure 1). Since the length of the extra-cortical, porous-coated body section was 4 cm, the cortical onlay pedicle was measured and marked 4–5 cm away from the very end of the bone segment. The cortical bone was divided into several parts along with the muscle attachments to maintain the blood supply. The cortical onlay pedicle autograft and allografts were affixed to this porous coating section with one or two wires. During this procedure, the periosteum was intended to maintain continuity with the remaining bone (Figure 2). The periosteum discontinued in several early cases, such as the cortical pedicle dividing into several separate parts. The potential advantages of this technique include the use of extracortical bone-bridging and ingrowth fixation to achieve biological fixation, which may share stress and prevent osteolysis by sealing off the critical region against the infiltration of ware particles. If the stem is shorter than the previous stem, the distal intramedullary cavity was grafted with allograft, which was expected to provide further augmentation for the prosthesis. Cemented short stem would preserve more bone stock and have immediate stability.

As per our protocol, patients were encouraged to have quadriceps exercise from the next day after the surgery. They were allowed 5–10 kg weightbearing for weeks 8 to 12 postoperatively, and then full weight bearing afterwards. The habitation depends on the status of bone healing. Patients were scheduled to take follow-up visits every 3 months in the first year, then every 6 months for 2 years, and then yearly. At each follow-up visit, patients receive both a physical examination as well as a radiographic evaluation. Physical examinations included pain, mobility, and knee range of motion (ROM) performed by the primary surgeon of the patient. Radiographic evaluations included tumor- and prosthesis-related complication monitoring. Radiographs were evaluated for bone ingrowth to the porous coating section in orthogonal views at final follow-up, as initially described by Coathup et al. [16]. The extracortical bone-bridging was assessed in four zones (the medial and lateral aspects on anteroposterior radiographs and the anterior and posterior aspects on lateral radiographs). A score of 1 represented extracortical bone-bridging (>5 mm thick and >2 cm long) in contact with the prosthesis surface in any one of the four (anteroposterior and mediolateral) zones. The score ranged from zero to four. The maximal score was 4, which represented the ingrowth of bone in all four zones. At final follow-up, the function was evaluated according to the score of the Musculoskeletal Tumor Society (MSTS) [17].

### Statistical Analysis

Follow-up time was expressed as the mean, median, standard deviation, and range. The 5-year and 8-year survivorship of the prosthesis was calculated using Kaplan–Meier analysis for all prosthesis failures. The time of any stage of aseptic loosening was recorded for both the primary prosthesis and the revision GMRS prosthesis. Two-tailed paired t test was used to compare the time of aseptic loosening between the primary prosthesis and the revision GMRS prosthesis. The time of aseptic loosening survival was calculated using the Kaplan–Meier method. We performed all statistical analysis using a commercially available software package (IBM SPSS Statistics for Mac, version 25, IBM, Armonk, NY, USA). A *p* value of <0.05 was considered significant in all tests.

## 3. Results

### 3.1. Survival of the Revision Prosthesis

**Figure 3:**
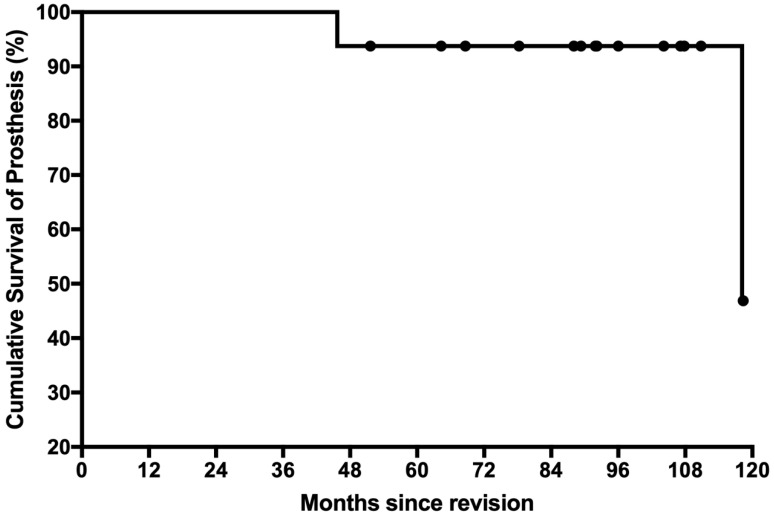
The cumulative survival of revision prosthesis is shown. Patients were censored at last follow-up.

No patients were lost to follow-up. No patients developed local nor systemic relapse. No patients died during the follow-up. The cumulative survival of all revision prosthesis was 94% (95%CI, 91–97%) at both 5 and 8 years (Figure 3). There were two prosthesis failures including one infection and one repeated aseptic loosening. The mean and median follow-up for the remaining 14 patients without prosthesis failure was 90 and 92 months, respectively (range, 52–118 months). One patient, aged 24, was primarily diagnosed as having conventional osteosarcoma in the distal femur. She received neoadjuvant chemotherapy, tumor resection and prosthesis replacement, and adjuvant chemotherapy. The aseptic loosening developed 51 months after the surgery and then she received GMRS revision. Twenty-eight months later, she developed prosthetic infection, necessitating the removal of the prosthesis without any sign of loosening. One patient, primarily with chondrosarcoma in the distal femur, developed repeated stage III aseptic loosening 118 months after the revision surgery. Since the patient was symptomatic, a repeated revision was performed using the same type of prosthesis without revising the tibia part.

### 3.2. Evaluation of Aseptic Loosening

The mean and median interval between the primary prosthesis replacement and the revision surgery were 81 and 73 months (range, 27–187 months). Except for the infection case, all the remaining 15 cases were evaluated using an X-ray of the last follow up. One patient primarily diagnosed with chondrosarcoma in the distal femur received their first GMRS revision surgery in another hospital. At the 33-month postoperative follow-up, she had good function without aseptic loosening. Then, she failed to participate in regular follow-up. She came back and complained of activity pain 118 months after the revision. The X-ray showed repeated aseptic loosening. This patient received re-revision and it has now been 22 months since then. The overall repeated aseptic loosening rate was only 6.3% (1/16).

**Figure 4:**
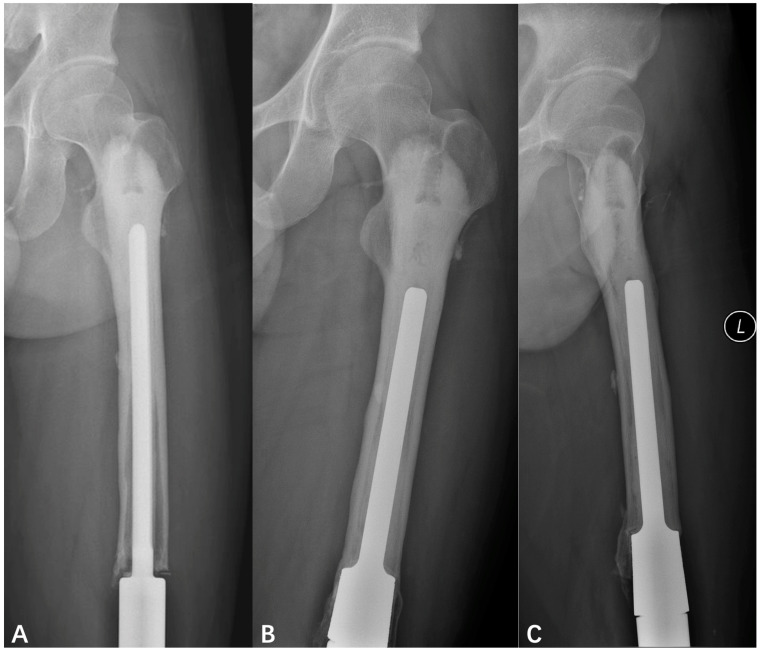
(**A**) AP radiographs of a 17-year-old man showed aseptic loosening at 75 months follow-up after prosthesis replacement. (**B**) The AP radiographs at 78-month follow-up post-revision showed no aseptic loosening and extracortical bone ingrowth in both the medial and the lateral aspects. (**C**) The lateral radiographs at 78-month follow-up post-revision showed no aseptic loosening and extracortical bone ingrowth in the posterior aspects but not the anterior aspect.

**Figure 5:**
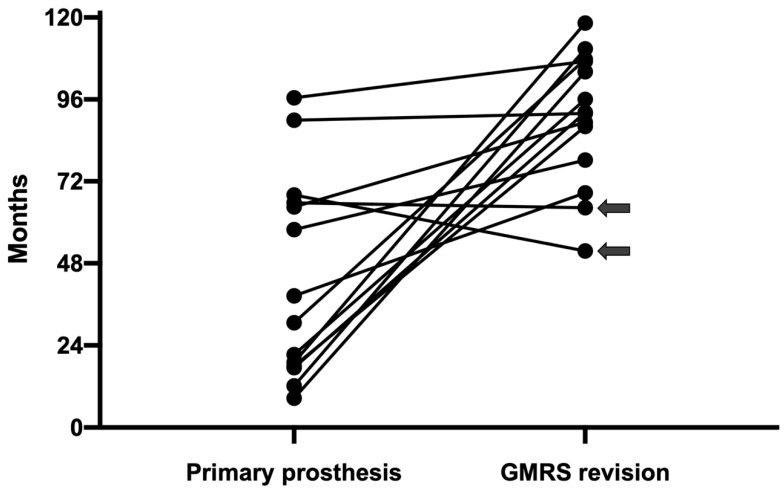
For each of the 14 cases there was a comparison made by connecting the time of aseptic loosening for the primary prosthesis and the follow up months for GMRS revision. Except for the two cases (as arrows marked) without sufficient follow up, all other 12 cases showed longer aseptic loosening free survival in favor of the revision prosthesis.

For the remaining 14 cases, at a median of 92 months (range, 52–118 months), no patient developed repeated aseptic loosening. In total, 85.7% (12/14) of the patients had much longer aseptic loosening free survival with revision prosthesis (90.6 ± 19.3 months) than with primary prosthesis (43.4 ± 29.7 months) with statistical significance (paired *t*-test, *t* = 4.297, p = 0.001) (Figure 4 and Figure 5). They were all evaluated for extracortical bone ingrowth using the X-ray results of the last follow up. The average extracortical bone ingrowth score was 3 (range, 0–4). The score was 4 (extracortical bone ingrowth seen at 4 zones including the medial and lateral aspects on anteroposterior radiographs, and the anterior and posterior aspects on lateral radiographs) for seven (50%) of the fourteen patients, 3 for three patients (21%), 2 for two patients (14%), 1 and 0 each for one patient (7%).

### 3.3. Functional Outcome of the Revision Prosthesis

Except for the infection case and the re-revision case (3 months after surgery), the remaining 14 cases were evaluated with MSTS score. At a median of 90 months (range, 52–118 months) after revision, the mean MSTS score was 27.7 (range, 24–30).

## 4. Discussion

Mega-prostheses play an increasingly role in reconstruction after musculoskeletal tumor resection. Despite improvements and innovations in materials and modular design, prosthesis failure remains higher than that for surface arthroplasty. Henderson et al. proposed five modes of failure for tumor prostheses, in which aseptic loosening was defined as type 2. The aseptic loosening failure for the lower extremity ranged from 7.7% to 18.8% [18,19,20,21,22], which was much higher than the overall reported incidence of aseptic loosening failure (4.7%) in all sites [6]. While many researchers have reported the outcomes of patients receiving primary prosthesis for musculoskeletal tumors, few authors have offered specific criteria to assess the aseptic loosening or reviewed the outcomes of patients receiving revision prosthesis [11,12,20]. Revisions for oncological prosthesis could be especially challenging due to the limited bone quantity and poor quality that the patients still possess. We reviewed our experience with oncologic prosthesis revisions and found a 94% cumulative survival of all revision prosthesis at both 5 and 8 years. Only one case developed aseptic loosening again, which made the repeated aseptic loosening rate as low as 6.3% (1/16).

The authors acknowledge several limitations to this research. First, this is a retrospective analysis of patients who had revisions for oncological mega-prosthesis. Some of the patients failed to take regular follow-ups, which made an understanding of the natural history of aseptic loosening development impossible. Second, our series of 16 patients is small. The evaluation for GMRS revisions requires a large population to validate its efficiency. However, our series is homogeneous with respect to the institution patients’ received treatment (only one institution), the indication (only for aseptic loosening), the anatomic location (the lower extremity), the revision prosthesis type, and the surgical techniques applied. Third, we lack a comparison group of revision patients using another prosthesis, such as one that is longer but thinner, which compromised the power of this research to a certain extent.

The contributing factors that lead to aseptic loosening have not been clearly defined yet. Kawai [23] and Unwin [24] reported that the resection percentage can predict aseptic loosening. For the distal femur and the proximal tibia, the longer the resection, the higher the risk of developing aseptic loosening. However, the opposite is true with regard to the proximal femur. Patrick et al. revealed only the bone:stem ratio as an independent risk factor for aseptic loosening. Patients with a larger stem size (φ 14.5 mm) presented longer aseptic loosening free survival than smaller ones (φ 10.7 mm). Our research supported the use of a large stem size (φ 13, 15 mm). Melissa et al. [12]. reported a relatively short stem prosthesis called CPS. With 90 months of median follow-up, the prosthesis failures included mechanical failure and deep infection; however, no aseptic loosening case was reported. In another report, the rate of aseptic failure was as low as 3.8% at 6.3 years for a CPS implant [25]. A research study about uncemented tumor endoprostheses at the knee reported that the aseptic loosening was only 2% (2/99) [26]. However, other complications including stem fracture and infection were higher than other reports. In the current series, there was one case of repeated aseptic loosening, and we speculated that the possible reason for this failure was poor extracortical bone ingrowth.

Extracortical bone-bridging and ingrowth in the porous-coated body sections were expected to result in biological fixation, not only sharing stress from the prosthesis to the host bone, but also preventing osteolysis by sealing off the junction area against the infiltration of wear particles [21]. Coathup et al. [16]. reported the distal femoral implants with a hydroxyapatite-coated grooved collar with survival rates of 88.9% at ten years and 91.8% after a mean duration of follow-up of 8.5 years. There was a trend for an increase in the survivorship of implants with osteointegration of the collar compared with those without osteointegration. In our research, we demonstrated a mean score of 3 in terms of f extracortical bone ingrowth, which may contribute and explain the low aseptic loosening rate of these revision surgeries. Functional outcomes in this series of revision patients were similar to those reported in the literature.

Alternative reconstructive options include structural allograft and allograft-prosthesis composite [27]. According to Muscolo’s report [28], the osteoarticular allograft survival was 78% at ten years. However, the 35% articular deterioration rate is worthy of further clinical research with long-term follow-up. Unfortunately, massive osteoarticular allograft was not available in many institutions, including ours.

## 5. Conclusions and Future Work

In summary, revisions for prosthesis aseptic loosening in oncological patients can be very challenging. We presented a reasonable option to choose when revising failed tumor prosthesis. The core of the prosthesis revision includes the preservation of the host bone stock, strong temporary fixation until bone healing, and use of cortical onlay pedicle to achieve biological fixation. However, on account of being limited by a lack of a large volume of homogenous aseptic loosening tumor patients, only prospective research would provide better evidence of this treatment. A larger study with a control group (e.g., longer stem without extra-cortical porous-coated body sections) and longer follow-up periods are warranted to prove the benefit of this prosthesis as an indication for revision.

## Figures and Tables

**Table 1:** Patients demographics.

Demographics of Patients	Numbers
Number of patients	16
Age (years; range)	28 (14–55)
Gender	
Male	9
Female	7
Tumor Diagnosis	
Conventional osteosarcoma	7
Giant cell tumor of bone	6
Chondrosarcoma	1
Chondroblastoma	1
Epitheliod haemangioendothelioma	1
Tumor locations	
Distal femur	10
Proximal tibia	4
Proximal femur	2
Primary prosthesis (knee)	
Rotating hinge knee	8
Constrained condylar knee	6

## Data Availability

The data that support the findings of this study are available from the corresponding author on reasonable request.
